# Supplementary material for: Limits to the Hall Effect and Other Nonreciprocal Effects in Three‐Dimensional Metamaterials
Source: Adv Mater. 2026 Sep 17;38(61):e17068. doi: 10.1002/adma.202517068 (PMC13726711; doi:10.1002/adma.202517068)
Supplement: Supplementary file 1 [file ADMA-38-e17068-s001.pdf]

Supporting Information: Limits to the Hall Effect and Other Nonreciprocal Effects in Three-Dimensional Metamaterials

Christian Kern[1,2*] and Graeme W. Milton[2†]

[1] *Department of Civil and Mechanical Engineering, Technical University of Denmark, Lyngby, Denmark*
[2] *Department of Mathematics, University of Utah, Salt Lake City, Utah, USA*

CONTENTS

SI. Extending the bounds to fully anisotropic materials 2
A. Hall tensor components 2
B. S-tensor components 3

SII. Analytic expressions 4
A. Two-phase bounds 4
B. Bounds on the diagonal Hall tensor components 4

* physics@chrkern.de
† graeme.milton@utah.edu

SI. EXTENDING THE BOUNDS TO FULLY ANISOTROPIC MATERIALS

In this section, we continue the discussion from Section 2.1 of the main text and show how the bounds on uniaxial metamaterials yield important implications for (generally) fully anisotropic metamaterials. The idea is to form uniaxial microstructures (involving several lamination steps and the formation of a polycrystal) from the fully anisotropic metamaterials. Applying bounds to these uniaxial microstructures then implies bounds on the effective properties of fully anisotropic metamaterials.

Specifically, on the basis of a first such microstructure, we demonstrate in Subsection SI A that the uniaxial bounds apply to fully anisotropic metamaterials upon making the replacements in Equation (17) of the main text, which we repeat here

$$\sigma_\perp^* \to \sqrt{\det\left(\boldsymbol{\sigma}^*\right)/\sigma_{33}^*} \text{ and } A_\parallel^* \to A_{33}^*.$$

On the basis of a second such microstructure, we establish in Subsection SI B that the uniaxial bounds also apply to fully anisotropic metamaterials upon making the alternative replacements in Equation (18) of the main text,

$$\sigma_\perp^* \to \sqrt{\sigma_{11}^*\sigma_{22}^* - {\sigma_{12}^*}^2} = \sqrt{\operatorname{Cof}\left(\boldsymbol{\sigma}^*\right)_{33}} \text{ and } S_\parallel^* \to S_{33}^*.$$

A. Hall tensor components

Consider a fully anisotropic metamaterial with effective zero magnetic-field (zmf) conductivity tensor $\boldsymbol{\sigma}^*$ and effective S-tensor $\boldsymbol{S}^*$. In addition to this metamaterial, we consider a copy that is mirrored at the x_1x_2-plane. This mirror copy has effective properties

$$\boldsymbol{\sigma}^{(*\mathrm{M})} = \begin{pmatrix} \sigma_{11}^* & \sigma_{12}^* & -\sigma_{13}^* \\ \sigma_{12}^* & \sigma_{22}^* & -\sigma_{23}^* \\ -\sigma_{13}^* & -\sigma_{23}^* & \sigma_{33}^* \end{pmatrix} \text{ and } \boldsymbol{S}^{(*\mathrm{M})} = \begin{pmatrix} S_{11}^* & S_{12}^* & -S_{13}^* \\ S_{21}^* & S_{22}^* & -S_{23}^* \\ -S_{31}^* & -S_{32}^* & S_{33}^* \end{pmatrix}. \tag{S1}$$

Next, we laminate the original metamaterial and its mirrored copy in equal proportions with layers perpendicular to the x_3-direction. Using the usual lamination equations and the perturbation expression (12), we obtain the following expressions for the effective conductivity tensor and the effective S-tensor,

$$\boldsymbol{\sigma}^{(*2)} = \begin{pmatrix} \sigma_{11}^* - \frac{(\sigma_{13}^*)^2}{\sigma_{33}^*} & \sigma_{12}^* - \frac{\sigma_{13}^*\sigma_{23}^*}{\sigma_{33}^*} & 0 \\ \sigma_{12}^* - \frac{\sigma_{13}^*\sigma_{23}^*}{\sigma_{33}^*} & \sigma_{22}^* - \frac{(\sigma_{23}^*)^2}{\sigma_{33}^*} & 0 \\ 0 & 0 & \sigma_{33}^* \end{pmatrix} \text{ and } \boldsymbol{S}^{(*2)} = \begin{pmatrix} S_{11}^* & S_{12}^* & 0 \\ S_{21}^* & S_{22}^* & 0 \\ 0 & 0 & \frac{\sigma_{13}^*}{\sigma_{33}^*}S_{13}^* + \frac{\sigma_{23}^*}{\sigma_{33}^*}S_{23}^* + S_{33}^* \end{pmatrix}. \tag{S2}$$

We now perform a second lamination. This time we laminate in equal proportions with layers perpendicular to the x_1-direction. The laminate is formed from the laminate from the previous step and a copy of it that is mirrored at the x_1x_3-plane. The effective properties of the resulting metamaterial are

$$\boldsymbol{\sigma}^{(*3)} = \operatorname{diag}\left(\sigma_{11}^* - \frac{(\sigma_{13}^*)^2}{\sigma_{33}^*}, \sigma_{22}^* - \frac{(\sigma_{23}^*)^2}{\sigma_{33}^*} - \left(\sigma_{12}^* - \frac{\sigma_{13}^*\sigma_{23}^*}{\sigma_{33}^*}\right)^2\left(\sigma_{11}^* - \frac{(\sigma_{13}^*)^2}{\sigma_{33}^*}\right)^{-1}, \sigma_{33}^*\right) \tag{S3}$$

$$\text{and } \boldsymbol{S}^{(*3)} = \operatorname{diag}\left(S_{11}^* + S_{21}^*\frac{\sigma_{13}^*\sigma_{23}^* - \sigma_{12}^*\sigma_{33}^*}{(\sigma_{13}^*)^2 - \sigma_{11}^*\sigma_{33}^*}, S_{22}^*, \frac{\sigma_{13}^*}{\sigma_{33}^*}S_{13}^* + \frac{\sigma_{23}^*}{\sigma_{33}^*}S_{23}^* + S_{33}^*\right). \tag{S4}$$

In the last step, we form a uniaxial polycrystal from this metamaterial. Specifically, we construct an extruded (columnar) version of a checkerboard. The squares of one of the two colors (e.g., the white squares) are made from the metamaterial formed in the previous step. In the squares of the other color (e.g., the black squares), the metamaterial is rotated by 90° about the x_3-axis. This polycrystal has effective properties

$$\sigma_\perp^{(*\mathrm{P})} = \sqrt{\det\left(\boldsymbol{\sigma}^*\right)/\sigma_{33}^*} \text{ and } A_\parallel^{(*\mathrm{P})} = A_{33}^*, \tag{S5}$$

which yields the desired conclusion.

B. S-tensor components

We now derive the second set of replacements under which the uniaxial bounds apply to fully anisotropic metamaterials. For this purpose, we will construct a second microstructure. As before, we denote the effective zmf conductivity tensor and the effective S-tensor of the fully anisotropic metamaterial as $\boldsymbol{\sigma}^*$ and $\boldsymbol{S}^*$, respectively. In contrast to the previous section, we initially assume that $\sigma_{12}^* = 0$. This assumption will later be relaxed.

First, we reflect the metamaterial at the x_2x_3-plane, yielding a mirrored version of the original metamaterial. The effective zmf conductivity tensor and the effective S-tensor of this mirrored version read

$$\boldsymbol{\sigma}^{(*\mathrm{M})} = \begin{pmatrix} \sigma_{11}^* & 0 & -\sigma_{13}^* \\ 0 & \sigma_{22}^* & \sigma_{23}^* \\ -\sigma_{31}^* & \sigma_{32}^* & \sigma_{33}^* \end{pmatrix} \text{ and } \boldsymbol{S}^{(*\mathrm{M})} = \begin{pmatrix} S_{11}^* & -S_{12}^* & -S_{13}^* \\ -S_{21}^* & S_{22}^* & S_{23}^* \\ -S_{31}^* & S_{32}^* & S_{33}^* \end{pmatrix}, \tag{S6}$$

respectively. Next, we laminate the original metamaterial and its reflected version in equal proportions with layers perpendicular to the x_1-direction. Thereby, we obtain a laminate with the following effective properties

$$\boldsymbol{\sigma}^{(*2)} = \begin{pmatrix} \sigma_{11}^* & 0 & 0 \\ 0 & \sigma_{22}^* & \sigma_{23}^* \\ 0 & \sigma_{32}^* & \sigma_{33}^* - \frac{(\sigma_{13}^*)^2}{\sigma_{11}^*} \end{pmatrix} \text{ and } \boldsymbol{S}^{(*2)} = \begin{pmatrix} S_{11}^* + \frac{\sigma_{13}^*}{\sigma_{11}^*} S_{31}^* & 0 & 0 \\ 0 & S_{22}^* & S_{23}^* \\ 0 & S_{32}^* & S_{33}^* \end{pmatrix}. \tag{S7}$$

We proceed with a second lamination step. Again we laminate the metamaterial obtained in the previous step with a copy of it that is mirrored at the x_1x_3-plane in equal proportions with layers perpendicular to the x_1-direction. The effective properties of the resulting laminate are

$$\boldsymbol{\sigma}^{(*3)} = \operatorname{diag}\left(\sigma_{11}^*, \sigma_{22}^*, \sigma_{33}^* - \frac{(\sigma_{13}^*)^2}{\sigma_{11}^*}\right) \text{ and } \boldsymbol{S}^{(*3)} = \operatorname{diag}\left(S_{11}^* + \frac{\sigma_{13}^*}{\sigma_{11}^*} S_{31}^*, S_{22}^*, S_{33}^*\right). \tag{S8}$$

As above, in the last step, we form a uniaxial polycrystal from this metamaterial. Specifically, we construct an extruded (columnar) version of a checkerboard. The squares of one of the two colors (e.g., the white squares) are made from the metamaterial formed in the previous step. In the squares of the other color (e.g., the black squares), the metamaterial is rotated by 90° about the x_3-axis. This polycrystal has effective properties

$$\sigma_{\perp}^{(*\mathrm{P})} = \sqrt{\sigma_{11}^* \sigma_{22}^*} \text{ and } S_{\parallel}^{(*\mathrm{P})} = S_{33}^*. \tag{S9}$$

To generalize beyond the original assumption $\sigma_{12}^* = 0$, we perform a rotation about the x_3-axis that eliminates this component. In the original coordinate system (i.e., prior to the rotation), this yields

$$\sigma_{\perp}^{(*\mathrm{P})} = \sqrt{\sigma_{11}^* \sigma_{22}^* - (\sigma_{12}^*)^2} = \sqrt{\operatorname{Cof}(\boldsymbol{\sigma}^*)_{33}} \text{ and } S_{\parallel}^{(*\mathrm{P})} = S_{33}^*, \tag{S10}$$

thereby confirming the validity of the alternative replacements for applying the uniaxial bounds to fully anisotropic metamaterials.

SII. ANALYTIC EXPRESSIONS

In this section, we will provide analytic expressions for the volume-fraction-dependent two-phase bounds in the effective parameter plane (Subsection SII A) as well as for the bounds on the diagonal components of the effective Hall tensor as a function of the conductivity contrast (Subsection SII B).

A. Two-phase bounds

We consider uniaxial metamaterials formed from two isotropic phases with conductivities σ_1, σ_2, with $\sigma_2 < \sigma_1$ and S-coefficients S_1, S_2. Our labeling of the bounds as "upper" and "lower" assumes that the phases are well-ordered, i.e., $S_1 > S_2$. For $S_1 < S_2$, the upper and lower bounds flip roles. As discussed in Section 5.1 of the main text, the effective properties of such metamaterials are confined by the bounds (47), which we repeat here,

$$|y_S + S_2| \leq |S_1 - S_2| \, \frac{y_{\sigma\perp} - \sigma_2}{\sigma_1 - \sigma_2} \text{ and} \tag{S11}$$

$$\left| y_S + \frac{S_1\sigma_2 + S_2\sigma_1}{\sigma_1 + \sigma_2} \right| \leq |S_1 - S_2| \, \frac{y_{\sigma\perp}}{\sigma_1 + \sigma_2}. \tag{S12}$$

These bounds form one line segment and two rays. The line segment connects the two points $(\sigma_2,\ -S_2)$ and $(\sigma_1,\ -S_1)$. The first and second rays start from the first and second points, respectively, with their extensions passing through $(0,\ -(S_1\sigma_2 + S_2\sigma_1)/(\sigma_1 + \sigma_2))$.

Through the inverse of the linearized y-transformation, (45) and (46), we obtain corresponding expressions for the volume-fraction-dependent bounds in the effective parameter plane, i.e., in the $\sigma_\perp^*$-$S_\parallel^*$-plane,

$$S_{\text{vf}}^{\text{lower}}(\sigma_\perp^*, f_1) \leq S_\parallel^* \leq S_{\text{vf}}^{\text{upper}}(\sigma_\perp^*, f_1). \tag{S13}$$

An analytic expression for the **upper bound**, which corresponds to the first ray in the y-plane, is given by

$$S_{\text{vf}}^{\text{upper}} = \frac{S_1\sigma_2 + S_2\sigma_1}{\sigma_1 + \sigma_2} - (S_1 - S_2)\frac{2\sigma_2(\sigma_1 - \sigma_\perp^*)^2 - f_2(\sigma_1 - \sigma_2)^2\sigma_\perp^*}{f_2(\sigma_1 - \sigma_2)^2(\sigma_1 + \sigma_2)}. \tag{S14}$$

As in the y-plane, the **lower** bound is composed of two sections. The first section corresponds to the straight-line segment in the y-plane, while the second section corresponds to the second ray. Analytic expressions for the first and second sections of the lower bound are

$$S_{\text{vf}}^{\text{lower}} = S_2 + (S_1 - S_2)\frac{f_1(\sigma_1 - \sigma_2)(\sigma_1 + 3\sigma_2)(\sigma_2 - \sigma_\perp^*) + 2(f_2\sigma_1 + f_1\sigma_2)(\sigma_2 - \sigma_\perp^*)^2 + f_1^2(\sigma_1 - \sigma_2)^2(\sigma_2 + \sigma_\perp^*)}{f_1 f_2(\sigma_1 - \sigma_2)^3} \text{ and} \tag{S15}$$

$$S_{\text{vf}}^{\text{lower}} = \frac{S_1\sigma_2 + S_2\sigma_1}{\sigma_1 + \sigma_2} + (S_1 - S_2)\frac{2\sigma_1(\sigma_2 - \sigma_\perp^*)^2 - f_1(\sigma_1 - \sigma_2)^2\sigma_\perp^*}{f_1(\sigma_1 - \sigma_2)^2(\sigma_1 + \sigma_2)}, \tag{S16}$$

respectively. The two sections of the lower bound meet at the point corresponding to the effective properties of the coated cylinder assemblage with phase 2 as the core phase. The corresponding effective zmf conductivity is

$$\sigma^{\text{CC2}} = \sigma_1 + \frac{2f_2\sigma_1(\sigma_2 - \sigma_1)}{2\sigma_1 + f_1(\sigma_2 - \sigma_1)}. \tag{S17}$$

Note that these expressions are significantly more complicated than the corresponding expressions in the y-plane. In particular, the latter hide the volume-fraction dependence of the bounds.

B. Bounds on the diagonal Hall tensor components

We continue the discussion from Section 5.3 of the main text. Recall that we are considering metamaterials formed from two isotropic phases with conductivities σ_1, σ_2, with $\sigma_2 < \sigma_1$ and Hall coefficients $A_1 \neq 0$ and $A_2 = 0$. As discussed in the main text, the range of any diagonal effective Hall tensor component, A_{ii}^*, increases with increasing conductivity contrast of the phases, σ_1/σ_2. For low conductivity contrasts, the pure phases are optimal. Above

$\sigma_1/\sigma_2 = 2$ enhancements and above $\sigma_1/\sigma_2 = 9$ sign-inversions are permitted by the bounds. More generally, A^*_{ii}/A_1 has to lie between an upper and a lower bound with the corresponding analytic expressions given by

$$A^{\text{upper}} = \begin{cases} 1 & \text{if } \sigma_1 \leq 2\sigma_2 \\ \frac{\sigma_1^2}{4\sigma_2(\sigma_1-\sigma_2)} & \text{if } \sigma_1 > 2\sigma_2, \end{cases}$$

$$A^{\text{lower}} = \begin{cases} 0 & \text{if } \sigma_1 \leq 9\sigma_2 \\ -\frac{\sigma_1^2(\sqrt{\sigma_1}-3\sqrt{\sigma_2})^2(\sigma_1-\sigma_2+2\sqrt{\sigma_1\sigma_2})}{4\sigma_2(\sigma_1-\sigma_2)(\sigma_1^2-6\sigma_1\sigma_2+\sigma_2^2)} & \text{if } \sigma_1 > 9\sigma_2. \end{cases} \tag{S18}$$

Note that, through the polycrystal argument discussed above, this result is not limited to uniaxial metamaterials. The upper bound is optimal for uniaxial metamaterials, as it is attained by a rank-1 laminate. Results for $A_2 \neq 0$ can, in principle, be obtained via a suitable translation, although this is more involved, as it is the antisymmetric part of the conductivity tensor and not of the resistivity tensor that is being shifted (translations of the resistivity tensor do not have the required invariance properties).
